# Altered Glucose Homeostasis Is Associated with Increased Serum Apelin Levels in Type 2 Diabetes Mellitus

**DOI:** 10.1371/journal.pone.0051236

**Published:** 2012-12-05

**Authors:** Maria Gisella Cavallo, Federica Sentinelli, Ilaria Barchetta, Carmine Costantino, Michela Incani, Laura Perra, Danila Capoccia, Stefano Romeo, Efisio Cossu, Frida Leonetti, Luciano Agati, Marco G. Baroni

**Affiliations:** 1 Endocrinology and Diabetes, Department of Medical Sciences, University of Cagliari, Cagliari, Italy; 2 Department of Internal Medicine and Medical Specialties, Sapienza University of Rome, Rome, Italy; 3 Department of Clinical Sciences, Sapienza University of Rome, Rome, Italy; 4 Department of Molecular and Clinical Medicine, Sahlgrenska Center for Cardiovascolar and Metabolic Research, University of Gothenburg, Gothenburg, Sweden; 5 Department of Cardiovascular Sciences, Sapienza University of Rome, Rome, Italy; University of Ulster, United Kingdom

## Abstract

**Background:**

Apelin is an adipokine that plays a role in the regulation of glucose homeostasis and in obesity. The relationship between apelin serum concentration and dysmetabolic conditions such as type 2 diabetes (T2D) is still controversial. Aims of our study are: 1) determine the circulating levels of apelin in a large cohort of Italian subjects with T2D, T1D and in non-diabetic controls; 2) identify putative metabolic determinants of modified apelin concentrations, in order to search possible mechanism of apelin control; 3) investigate changes in apelin levels in response to sharp modifications of glucose/insulin metabolism in T2D obese subjects before and 3 days after bariatric surgery.

**Methods:**

We recruited 369 subjects, 119 with T2D, 113 with T1D and 137 non-diabetic controls. All subjects underwent a complete clinical examination, including anthropometric and laboratory measurements. Serum apelin levels were determined by EIA (immunoenzyme assay).

**Results:**

Patients with T2D had significantly higher serum apelin levels compared to controls (1.23±1.1 ng/mL *vs* 0.91±0.7 ng/mL, P<0.001) and to T1D subjects (0.73±0.39 ng/mL, P<0.001). Controls and T1D subjects did not differ significantly in apelin levels. Apelin concentrations were directly associated with fasting blood glucose (FBG), body mass index (BMI), basal Disposition Index (DI-0), age, and diagnosis of T2D at bivariate correlation analysis. Multiple regression analysis confirmed that diagnosis of T2D, basal DI-0 and FBG were all determinants of serum apelin levels independently from age and BMI. Bariatric surgery performed in a subgroup of obese diabetic subjects (n = 12) resulted in a significant reduction of apelin concentrations compared to baseline levels (P = 0.01).

**Conclusions:**

Our study demonstrates that T2D, but not T1D, is associated with increased serum apelin levels compared to non-diabetic subjects. This association is dependent on impaired glucose homeostasis, and disappears after bariatric surgery, providing further evidence regarding the relationship between apelin and the regulation of glucose metabolism.

## Introduction

Insulin resistance is a major characteristic of type 2 diabetes mellitus (T2D) and is often linked to obesity [1]. In combination, these events increase the risk of cardiovascular diseases and obesity-associated morbidity. Excess in adipose tissue plays a central role in the induction of insulin-resistance.

In the last decade, many different studies demonstrated the existence of a number of adipocyte-derived secretory factors (“adipokines”) which take part in the control of body energy homeostasis and glucose metabolism [2]. Among them, a new peptide has been recently identified, named apelin (also known as APJ receptor ligand) [3]. Boucher *et al.* demonstrated that apelin is produced and secreted by both human and mouse white adipose tissue, acting therefore as an adipokine [4]. Apelin mRNA is detectable in non-differentiated preadipocytes and its production increases 4-fold upon differentiation of the fat cells, as previously found for adiponectin and leptin [4]. In humans, apelin gene is widely expressed in adipose tissue, heart, stomach, placenta and breast, as well as in different brain areas, suggesting an important role of this molecule also in the central regulation of metabolic pathways [5].

Native preproapelin exists as a dimer of 77 amino acids that is cleaved into active forms of C-terminal fragments, including apelin-36, apelin-17, apelin-13, and the post-translationally modified (Pyr1)apelin-13 and apelin-12 [6]. All of these predicted isoforms have been shown to be present in vivo. Apelin-12 is the smallest C-terminal fragment to bind and activate the apelin receptor [7], and any apelin fragment containing this 12 amino acid core maintains all bioactivity.

Findings from several studies suggest that apelin treatment during insulin resistance triggers a number of coordinated beneficial effects, including reduction of hyperinsulinemia and adiposity, and stimulation of glucose uptake and fuel consumption [8]. Insulin resistance in muscle is characterized by impaired glucose uptake, reduced glycogen synthesis, insufficient fat oxidation, fat accumulation and cellular stress. In skeletal muscle apelin has been shown to improve the overall insulin-sensitivity, both in vitro and in animal models [9]. In adipose tissue apelin infusion in apelin^−/−^ mice decreased adiposity and FFAs and also glycerol levels, suggesting a role for apelin in the regulation of lipolysis [10]. However, in human adipose tissue explants or in human isolated adipocytes, apelin had no effect on basal or isoproterenol-stimulated lipolysis [11]. Finally, in pancreas apelin was also shown to inhibit both glucose-induced and glucagon- like peptide 1 (GLP-1) stimulated insulin secretion in INS-1 cells [12], indicating that apelin acts as a regulator of insulin-secretion.

In humans, evidences on apelin regulation in presence of impaired glucose metabolism are still controversial. Some studies found increased apelin levels in very small populations of obese patients with impaired glucose tolerance or T2D [13]; [14]. On the opposite, other authors surprisingly reported low apelin levels in obese subjects with newly diagnosed T2D compared to non-diabetic individuals [15]; [16]. In a small sample of children with type 1 diabetes apelin levels were reported to be increased compared to healthy controls [17]. In the only large study in patients with gestational diabetes no difference was found in apelin levels between patients and control women [18]. Thus more consistent data are warranted.

Bariatric surgery could be viewed as a model of diabetes remission. To our knowledge, only one study compared serum apelin concentrations before and after bariatric surgery, showing a significant reduction only in patients affected by impaired glucose regulation or T2D before surgery [13].

Thus, aims of our study are: 1) to determine serum apelin levels in a large cohort of Italian subjects with T2D, T1D and in non-diabetic controls; 2) to identify putative metabolic determinants of modified apelin concentrations, in order to explore possible mechanism of apelin control; 3) to explore changes in apelin levels in response to sharp modifications of glucose/insulin metabolism in T2D obese subjects before and 3 days after bariatric surgery.

## Methods

### Ethics Statement

This study was reviewed and approved by the Ethics Committee of Policlinico Umberto I, Sapienza University of Rome and conducted in conformance with the Helsinki Declaration.

Written consent was obtained from all patients before the study.

### Population

For these purposes, we recruited 369 subjects, 119 affected by T2D (mean age±SD: 61±10 years), 113 with T1D (mean age±SD: 25±14 years), and 137 non-diabetic controls (mean age±SD: 49±10 years), among subjects attending the Internal Medicine out-patient clinics of Sapienza University of Rome. All subjects had a complete work-up including clinical examination, anthropometric measurements and laboratory tests.

### Laboratory Determinations

Study population underwent fasting blood sampling to assess FBG, glycosylated hemoglobin (HbA1c), total cholesterol, HDL-cholesterol, triglycerides, aspartate aminotransferase (AST), alanine aminotransferase (ALT), nitrogen and creatinine by standard laboratory methods. Insulin was measured by radio-immuno-assay (ADVIA Insulin Ready Pack 100, Bayer Diagnostics, Milan, Italy), with intra- and inter-assay coefficients of variation <5%. Low-density lipoprotein (LDL) cholesterol value was obtained using Friedwald formula. The homeostasis model assessment of insulin resistance (HOMA-IR) was calculated as previously described [19]. Metabolic Syndrome (MS) was defined according to modified NCEP ATP-III criteria [20] and diabetes mellitus according to ADA 2009 criteria [21].

Basal disposition index (DI-0), which gives an adjusted measure of insulin sensitivity according to the HOMA of insulin resistance (HOMA-IR) is calculated by using the formula DI-0 = HOMA-B* (1/HOMA-IR) [22]. The DI is a measure of the ability of the beta-cells to compensate for insulin resistance. It can be considered a measure of the functionality of the pancreas and can predict the normal beta-cell response adequate for any degree of insulin resistance [23]. In diabetes, beta-cells are unable to respond adequately to insulin-resistance, thus determining the appearance of impaired glucose regulation and altering the Disposition Index.

Apelin-12 levels were measured by a non-selective enzyme-linked immunosorbent assay (ELISA) kits (EK-057-23; Phoenix Pharmaceutical, Inc. Belmont, CA, USA) on sera frozen immediately after separation and stored at −25°C. The Apelin-12 EIA Kit presents 100% cross-reactivity with human Apelin-12, Apelin-13 and Apelin-36. The sensitivity of the technique was 0.06 ng/mL and the intra-assay and inter-assay CV% reported by the manufacturer were 5–10% and <15%, respectively.

### Statistics

SPSS version 17 statistical package was used to perform the analyses. These included Student’s t test for normally-distributed variables, Mann-Whitney non-parametric independent sample test and χ^2^ test for categorical variables, as appropriate. Correlations were estimate by Spearman’s rho non parametric test. The Wilcoxon’s rank test for paired samples was used to compared clinical and biochemical parameters of T2D patients before and after bariatric surgery.

A multiple liner regression analysis was performed to investigate independent association between serum apelin levels (dependent variable) and selected variables that had p-values <0.05 in univariate analysis (sex and age were also included). P-values <0.05 were considered statistically significant with a confidence interval of 95%.

We performed a sample size calculation based on the standard deviation (SD) of apelin levels in controls (SD = 0.70 ng/mL). If the true difference in the experimental and control means was 0.30, we needed to study 86 cases affected by diabetes vs 86 subjects without diabetes to be able to reject the null hypothesis that the population means of the patients and the controls were equal with probability (power) 0.80. The Type 1 error probability associated with this test is 0.05.

## Results

### Study Subjects

Clinical and metabolic characteristics of study population are summarized in Table 1. Briefly, patients affected by T2D had significantly higher age, FBG, fasting insulin, BMI, HbA1c, HOMA-IR, total-cholesterol, LDL-cholesterol, transaminases, systolic and diastolic blood pressure and lower HDL-cholesterol compared to non-diabetic control, as expected. T1D subjects had significantly higher glucose, HbA1c, and lower age, BMI, triglycerides compared to non-diabetic controls.

**Table 1:** Clinical and biochemical characteristics of study population.

	Non-diabetic controls (n = 137)	T2D(n = 119)	P	T1D(n = 113)	P*vs controls
**Age (years)**	49±11	61±10	<0.001	25±14	<0.001
**Sex (M/F)**	65/72	75/43	0.008	64/49	0.164
**Weight (Kg)**	74.3±16.3	82.2±18.9	0.005	67.6±12.6	0.022
**BMI (Kg/m^2^)** ^a^	27.4±5.6	30.4±4.5	<0.001	22.2±7.1	<0.001
**Waist (cm)**	94.5±16.5	104.6±13	<0.001	85±15.4	0.084
**SBP (mmHg)**	121±18	133±15	0.001	117±15	0.006
**DBP (mmHg)**	79±8	82±10	0.020	76±11	0.164
**Fasting blood glucose (mg/dL)**	96±16	141±45	<0.001	138.6±69.5	<0.001
**Fasting blood insulin (µU/mL)**	21.7±25	37.7±33	0.001	–	–
**DI-0**	66.1±43.5	26.1±27.9	<0.001	–	–
**HbA1c (%)**	5.4±0.4	7.1±1.5	0.001	7.1±1.6	0.001
**HOMA-IR (U)**	5.2±7	12.9±11	<0.001	–	–
**Total-cholesterol (mg/dL)**	204±43	281±44	<0.001	197.1±39.5	0.240
**HDL (mg/dL)**	54.6±15	49±20	0.001	58.4±18.3	0.383
**LDL (mg/dL)**	126±37	108±42	0.007	116.7±31	0.148
**Triglycerides (mg/dL)**	125±75	155±88	0.003	113.3±143	0.021
**AST (IU/L)**	21±8	25±13	0.04	20±7	0.507
**ALT (IU/L)**	24±14	33±19	<0.001	21.6±10	0.543
**Diabetes’ duration (years)**	–	7.97±7.95	–	6.4±7.1	–
**Apelin (ng/mL)**	0.91±0.7	1.23±1.1	<0.001	0.73±0.39	0.077

Data are expressed as mean ± standard deviation. Mann-Whitney non-parametric independent sample test was applied. P values <0.05 are considered significant. Abbreviations : BMI, body mass index; SBP, systolic blood pressure; DBP, diastolic blood pressure; DI-0 basal disposition index; HbA1c, glycated hemoglobin A1c; HOMA-IR, homeostatic model assessment of insulin resistance; HDL, high density lipoprotein; LDL, low density lipoprotein; AST, aspartate aminotransferase; ALT, alanine aminotransferase.

Serum apelin levels were significantly higher in T2D patients compared to controls (1.23±1.1 ng/mL vs 0.91±0.7 ng/mL, P<0.001) and to T1D subjects (0.73±0.39 ng/mL P<0.001). No difference was observed in apelin levels between controls and T1D subjects (P = NS) (Table 1).

### Correlation of Apelin with Metabolic and Clinical Parameters

Spearman’s correlation analyses in our population of T2D and non-diabetic controls (without T1D) demonstrated that serum apelin levels were directly associated with FBG, BMI, age and diagnosis of T2D, and negatively associated with basal Disposition Index (DI-0), whereas no association was found with other clinical and metabolic parameters (Table 2). In T1D apelin levels negatively correlated with HbA1c and positively with duration of the disease (data not shown).

**Table 2:** Correlation between serum apelin levels and clinical-biochemical parameters in study group (T2D and control subjects) with the other anthropometric and biochemical variables.

	r	P
**Age**	0.188	**0.007**
**Sex**	−0.020	0.751
**Weigth**	0.043	0.583
**BMI**	0.163	**0.036**
**Waist**	0.095	0.305
**SBP**	0.064	0.419
**DBP**	−0.029	0.715
**Fasting blood glucose**	0.152	**0.027**
**Fasting blood insulin**	0.087	0.265
**DI 0**	−0.209	**0.015**
**HbA1c%**	0.129	0.218
**HOMA-IR**	0.147	0.085
**Total-cholesterol**	0.011	0.879
**HDL-cholesterol**	0.093	0.195
**LDL-cholesterol**	0.050	0.520
**Triglycerides**	0.018	0.806
**AST**	0.116	0.112
**ALT**	0.107	0.140
**Diabetes’ duration**	0.057	0.639
**T2D**	0.183	**0.003**
**MS**	0.072	0.288

Spearman’s correlation coefficient. P values <0.05 are considered significant.

Abbreviations: BMI, body mass index; SBP, systolic blood pressure; DBP, diastolic blood pressure; DI-0 basal disposition index; HbA1c, glycated hemoglobin A1c; HOMA-IR, homeostatic model assessment of insulin resistance; HDL, high density lipoprotein; LDL, low density lipoprotein; AST, aspartate aminotranferase; ALT, alanine aminotransferase; MS, metabolic syndrome.

### Multivariate Analysis of Apelin Determinants

Multiple regression analysis with all the significant variables from the Spearman’s correlation analyses confirmed that diagnosis of T2D, basal Disposition Index and fasting plasma glucose were all determinants of serum apelin levels independently from age, and BMI (P = 0.04) (Table 3). Adjustment for sex did not change the result.

**Table 3:** Multiple linear regression analysis. Serum apelin concentration is the dependent variable.

	Standardized Regression Coefficients	t value	P
**Age**	−0.072	−0.768	0.444
**BMI**	0.094	1.091	0.277
**FBG**	−0.072	−0.671	0.047
**DI 0**	−0.517	−2.056	0.043
**T2D**	0.286	2.103	0.038

P values <0.05 are considered significant. Apelin levels was log10 transformed before the analysis.

Abbreviations: BMI, body mass index; FBG, Fasting blood glucose, DI-0 basal disposition index.

In order to further investigate the relative influence of BMI on serum apelin levels, we stratified our whole population (T2D and controls) in three groups of normal-weight (BMI = 20–24.9 Kg/m^2^), overweight (BMI: 25–29.9 Kg/m^2^) and obese (BMI>30 Kg/m^2^) subjects and found significantly increased apelin levels in presence of higher BMI (0.82±0.58 ng/mL in normal-weight, 1.01±0.87 ng/mL in overweight, 1.3±1.2 ng/mL in obese subjects, trend test P = 0.03). Then, the study population was grouped according to the presence/absence of T2D and we observed that, among the obese subgroup (BMI>30 Kg/m^2^), patients with T2D had significantly higher apelin levels than non-diabetic subjects (1.62±1.43 ng/mL vs 0.97±0.78 ng/mL, P = 0.015), thus confirming the strong independent association of diabetes with apelin levels.

### Antidiabetic Treatment and Apelin Levels

We also analysed the possible influence of antidiabetic treatment on apelin levels. Seventy-three percent (n = 87) of our T2D patients were treated with oral antidiabetic drugs, with 77% of them receiving metformin alone or in combination, and only a few (n = 14) were insulin-treated. Apelin levels were not significantly different between any of the antidiabetic regimens (data not shown).

### Apelin Levels and Bariatric Surgery

Finally, we explored serum apelin concentration changes after bariatric surgery in a subgroup of obese patients affected by T2D (n = 12), who underwent laparoscopic sleeve gastrectomy for weight reduction. Three days after surgery, when metabolic changes are already evident but before any significant reduction in weight and BMI, apelin levels were significantly decreased compared to baseline (2.03±2.5 vs 0.86±0.44 ng/mL, P = 0.013, respectively). T2D obese subjects showed also a significant reduction in fasting blood glucose (from 145.6 to 110.2 mg/dL, P<0.008), and an increase in DI-0 (22.3±20.3 vs. 46.5±32.02, P<0.008). Nine (75%) of these patients were without diabetes already after 3 days (Table 4 summarizes clinical characteristics of this population before and after the intervention). No significant difference was observed in apelin levels before and after bariatric surgery (1.83±0.54 vs 1.63±0.31 ng/mL, P = 0.279, respectively) in those 3 subjects that remained with type 2 diabetes, with fasting blood glucose 165±28 mg/dL.

**Table 4:** Clinical and biochemical characteristics of T2D patients before and after bariatric surgery.

	Pre-surgery (n = 12)	3 days Post-surgery(n = 12)	P-value
**Age (years)**	49±10	–	–
**Sex (M/F)**	5/7	–	–
**BMI (Kg/m^2^)**	40.8±8.9	39.5±8.0	0.051
**Fasting blood glucose (mg/dL)**	145.6±46.6	110.2±33.6	0.008
**Fasting blood insulin (µU/mL)**	33.9±25.4	24±18.9	0.367
**HOMA-IR (U)**	13.04±8.5	8.35±7.5	0.096
**DI 0**	22.3±20.3	46.5±32.02	0.008
**Apelin (ng/mL)**	2.03±2.5	0.86±0.44	0.013

Results are shown as Mean±SD. Non-parametric Wilcoxon Signed Rank test applied. P values <0.05 are considered significant.

To analyze if the reduction of apelin persisted after a longer period of observation, we evaluated apelin levels in sera from a subgroup of our diabetic obese subjects (n = 10), 6 months after bariatric surgery. Apelin levels remained low, showing only a modest non-significant (P = 0.55) increase compared to 3 days levels, (1.20±0.41 vs 0.86±0.44 ng/mL, respectively). This increase followed the modest increase observed in fasting glucose levels (from 110 to 119 mg/dL, P = NS). None of these subjects returned diabetic, but some (n = 3) showed fasting levels close to the diabetic threshold. Moreover, apelin levels after 6 months did not show any relationship with the important reduction in BMI observed in these patients (from 40.8 to 31.5 Kg/m^2^ P<0.001).

## Discussion

The results of this study demonstrate, in a large cohort of Italian subjects, that patients with type 2 diabetes have significantly increased serum apelin levels compared to non-diabetic individuals. The relationship between T2D and apelin is dependent on the presence of alterations in glucose homeostasis but independent from BMI and other metabolic abnormalities.

We also show for the first time that a key determinant of serum apelin levels is the basal disposition index, a surrogate measure of the two major defects in type 2 diabetes, defective insulin secretion and impaired insulin-sensitivity. Further support on this relationship is given by the low levels of apelin observed in type 1 diabetic subjects, where insulin secretion is absent and insulin-resistance is usually not present. Also Dray observed increased apelin concentrations in insulin-resistant type 2 diabetic patients, and this increase correlated positively with insulin levels. The compensatory role of apelin in glucose homeostasis was also confirmed by the phenotype of apelin null mice that are hyperinsulinemic and insulin resistant. It was shown that apelin treatment in these insulin-resistant obese-mice improves insulin sensitivity [24]. All together, these data may indicate that the increased apelin levels that we observe in T2D patients could represent a compensatory mechanism to reduce both insulin resistance and impaired insulin-secretion. Alternatively, the apelin increase may be due to a so called apelin-resistance, driven by yet unknown mechanism determining this effect.

In our study we do not observe significant correlation between apelin-12 concentration and HOMA-IR. The absence of association with HOMA-IR may be explained by the observation that the strongest association is found with type 2 diabetes, were both insulin resistance and impaired insulin secretion are present and necessary. Thus, it is possible that HOMA-IR alone cannot determine the increase of apelin in diabetic subjects. The disposition index, representing both defective insulin secretion and sensitivity, describes more effectively the presence of both defects.

In a previous study Boucher *et al.* showed a significant increase in apelin plasma concentrations in different mouse models of obesity associated with hyperinsulinemia, but not in the non-hyperinsulinemic obese mouse. Furthermore, in non-diabetic obese patients both plasma apelin and insulin levels were significantly higher than lean controls, suggesting that insulin could influence blood concentrations of apelin [4]. In our study we were unable to find a significant correlation between apelin and insulin levels, but we should point out that only 15% of our T2D patients were untreated, with most of these subjects taking metformin and/or sulphonylureas or insulin.

Consistent with previous reports, we observed a significant increase in apelin levels with increasing BMI. We also observed that obese patients with type 2 DM had significantly higher apelin levels than non-diabetic obese subject (1.62±1.43 ng/mL vs 0.97±0.78 ng/mL, P = 0.015) confirming that increased apelin levels are directly associated with the presence of diabetes rather than obesity itself.

With regards to the association between apelin and diabetes, in a very small study, Dray *at al.* also found increased apelin plasma levels in diabetic patients (n = 12) compared to controls (n = 11), in line with our observations in a larger cohort [14]. In contrast, when apelin was measured in untreated T2D at the time of diagnosis, its levels were found to be reduced compared to healthy controls [15], [16]. It may be postulated that a longer duration of diabetes might worsen insulin resistance and secretion, further influencing apelin levels. Newly diagnosed diabetic patients are in the early stage of their natural history, when the metabolic defects are not fully expressed, as shown by the possible reversal of T2D with lifestyle measures. Therefore we suggested that differences in diabetes duration may underlie discrepancies between studies.

In patients with type 1 diabetes we observed levels of apelin comparable to non-diabetic controls. In the only other study in children with T1D [17] the authors observed significantly increased apelin levels compared to age-matched controls. In an attempt to explain this discrepancies, we noticed that in this study the reported standard deviations for apelin levels were 2–4 times higher than those found in other studies, thus with a measurement not comparable, and that the sample was much smaller and younger than in our study.

In our study, a subgroup (n = 12) of our obese type 2 diabetes subjects underwent laparoscopic sleeve gastrectomy for weight reduction. Three days post surgery, a significant reduction in apelin, fasting blood glucose and basal disposition index was observed, together with a concomitant very small, non-significant, BMI reduction. Moreover, insulin levels after bariatric surgery showed a strong decrease, although not significant. The choice of selecting samples 3 days after laparoscopic sleeve gastrectomy was taken in order to observe possible changes in apelin levels before any significant weight loss would occur. Therefore, given that there were no significant differences in BMI at 3 days post-surgery, we could speculate that apelin reduction may be due to the changes observed in insulin sensitivity and insulin secretion (measured by the Disposition Index) after bariatric surgery rather than to a reduction of the adipose tissue mass. A further possible explanation may be ascribed to the changes in blood glucose, with remission of diabetes in 75% of the cases after 3 days post-surgery. Although the study was not designed to evaluate long-term effects, we were able to analyse 10 samples from the bariatric surgery group that were collected after 6 months. Lower levels of apelin compared to baseline persisted after six months from surgery and were not related to the highly significant reduction in BMI observed.

A recent association between apelin and TNF-alpha has been reported [25] in subjects with the metabolic syndrome. Also, Daviaud *et al.* [26] reported a significant association between TNF-alpha and apelin in adipose tissue of a mouse model of obesity. Since TNF-alpha is a marker of low-grade inflammation, which is present in the insulin-resistant state, it could be also seen as a marker of insulin-resistance, as it is for apelin. Although inflammation may still be present, we hypothesize that the dramatic metabolic changes observed in our subjects are probably the main determinants of apelin changes.

Soriguer *et al*. found increased apelin levels in morbidly obese patients with type 2 diabetes and a correlation between pre-bariatric surgery apelin plasma levels and BMI in diabetic patients [13]. Moreover, after surgery, they could observe a significant decrease of apelin levels only in the morbidly obese subjects with impaired fasting glucose or diabetes, a decrease that followed the changes in blood glucose and insulin sensitivity. These results are in line with our observation that BMI is not an independent determinant of high apelin levels, whereas diabetes is directly associated to increased apelin, regardless to other metabolic parameters.

The surgical technique that was used in our patients was the Laparoscopic Sleeve Gastrectomy, that deeply alters the complex balance of gastrointestinal hormones, such as ghrelin, leptin, GLP-1, peptide YY (PYY) and pancreatic polypeptide (PP) [27], [28]. Changes in these peptides appear to be critical for improving the response to insulin. However, we cannot find experimental data linking directly these peptides to apelin. It is possible that the whole system, greatly modified by the surgical intervention, acts to improve insulin-sensitivity and, as a consequence, to reduce apelin levels.

Based on published data and on our own observations, the pharmacological utility of apelin appears potentially useful. So far, the only evidences available are in animal models, where apelin administration, both long and short-term, improves insulin sensitivity and glucose regulation in different animal model [24], [9]. Furthermore, given the pleiotropic effects on numerous organs and tissues that are ascribed to apelin, the consequence of chronic or acute apelin administration in humans on cardiovascular functions, on central nervous system, and on glucose or lipid metabolism needs to be evaluated. The development of ligands agonists or antagonists versus apelin receptor are certainly warranted.

In conclusion, we investigated serum apelin levels in a large cohort of Italian subjects with T2D compared to non-diabetic controls and to patients with T1D. We demonstrated that T2D is a determinant of increased circulating apelin levels independently from the concomitant presence of obesity and other metabolic alterations. Apelin is increased in relation to the worsening of insulin-resistance/insulin-secretion, as in the diabetic status, and this increase has been suggested as a possible compensatory mechanism. Also, apelin levels, 3 days after bariatric surgery, significantly decreased together with an improved metabolic profile and independently from weight loss. This reduction persisted after six month, and was still unrelated to weight loss. Further studies are warranted to clarify the pathophysiological role of apelin in whole body energy homeostasis and in metabolic diseases.
